# Initial Experience with a Novel Pericardiocentesis Training Simulator Incorporating a Three-dimensional Printed Cardiac Model

**DOI:** 10.19102/icrm.2021.120603

**Published:** 2021-06-15

**Authors:** Sam L. Tsai, Stephen P. Seslar

**Affiliations:** ^1^Geisel School of Medicine, Dartmouth College, Hanover, NH, USA; ^2^Division of Pediatric Cardiology, Department of Pediatrics, Seattle Children’s Hospital, University of Washington, Seattle, WA, USA

**Keywords:** Medical simulation training, perforation, pericardial effusion, simulator, tamponade

## Abstract

Pericardial effusion is a rare but serious complication in cardiac electrophysiology procedures. To avoid progression to acute tamponade and reduce the risk of adverse patient outcomes, emergent pericardiocentesis is often necessary. The conduct of more pericardiocentesis training may further mitigate this risk. However, teaching and practice opportunities are rare, creating the need for pericardiocentesis simulators. While various pericardiocentesis simulators exist, their applications have been limited; further, commercial simulators are anatomically realistic but can be expensive. As such, cheaper homemade simulators have been developed, yet these may lack the cardiac anatomical features for a high-fidelity simulation or may be overly complex to assemble. The purpose of this study is to report initial findings from a pericardiocentesis simulator that incorporates a three-dimensional (3D) cardiac model that is economical, simple to assemble, and anatomically accurate. A 3D-printed cardiac model was printed from a computed tomography file. The model was fitted with a latex balloon-in-a-balloon pericardium filled with colored saline and placed in an ultrasound-compatible gelatin mold to create a pericardiocentesis simulator. The simulator was then tested with experienced and novice trainees at an academic hospital. A total of 10 participants (four interventional cardiology faculty members and six cardiology fellows) performed simulated pericardiocentesis using the simulator and completed a questionnaire to evaluate the model’s features and usefulness. The overall feedback regarding this novel simulation approach was positive and the model exhibited important anatomical features to accurately simulate ultrasound-guided pericardiocentesis. All participants were able to successfully insert the needle into the pericardial space and all but one successfully placed the pericardial drain. Survey results indicated that the model was largely perceived as useful for training. This work suggests incorporating a 3D-printed cardiac model into a gelatin mold results in a simple and inexpensive yet high-fidelity pericardiocentesis simulation experience. This novel approach may be useful for teaching pericardiocentesis in an academic hospital.

## Introduction

Cardiac perforation with pericardial effusion/tamponade is the most common serious complication of catheter ablation^1^ and the primary cause of cardiac ablation-related mortality.^2,3^ Treatment of a pericardial effusion/tamponade event generally involves the reversal of anticoagulation and percutaneous pericardiocentesis with the placement of a pericardial drain.^4,5^ Rapid and skilled management of the effusion is necessary to avoid progression to acute cardiac tamponade, in which a patient’s hemodynamics are compromised.^6^

Teaching and practice opportunities are rare and suboptimal during clinical procedures. As such, pericardiocentesis training simulators have been developed. Unfortunately, their application has been limited. Commercially manufactured models are anatomically accurate but can cost up to US$18,000.^7^ In response to this, various groups have created inexpensive models.^8–11^ However, while these models are simple, important anatomic landmarks, such as cardiac chambers, have often been missing, resulting in a lower-fidelity experience. More recently, Baribeau et al.^8^ reported on a pericardiocentesis simulator that incorporates a three-dimensional (3D) printed cardiac model, but this simulator may be more complicated than is necessary to achieve a high-fidelity pericardiocentesis simulation experience. The purpose of this study is to report our initial findings from the use of a simple, inexpensive pericardiocentesis simulator that incorporates a 3D-printed heart model in an inexpensive ultrasound-compatible gelatin mold.

## Methods

The Seattle Children’s Institutional Review Board (IRB) assessed this study, deeming it not to be human subjects research and thereby exempt from IRB oversight.

### Three-dimensional–printed heart model

**Figure 1:**
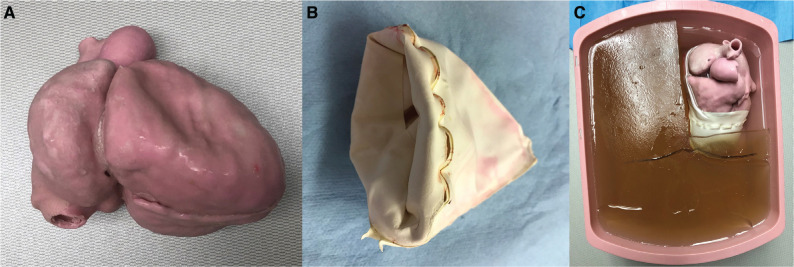
A 3D-printed silicone heart and pericardium model in a gelatin block. **A:** The 3D-printed cardiac phantom—the right atrial and right ventricular free walls can be seen. **B:** The balloon-in-a-balloon pericardial sac design is depicted. **C:** The heart model is placed where the gelatin section was removed. The uncolored water is allowed to fill the space around the heart as well as the inside chambers of the heart to aid with ultrasound-wave transmission. 3D: three-dimensional.

Using established and proprietary rapid prototyping techniques, we created an anatomically accurate 3D physical heart model based on magnetic resonance imaging data. DICOM images were segmented using 3D Slicer, an open-source volume-rendering software (www.slicer.org). The resultant .stl file was exported to a 310 3D printer machine with a proprietary hybrid powder and ZP 160 binder (Z Corporation, Rock Hill, SC, USA). Models were constructed using standard print parameters, resulting in a chalk-like porous scaffold whose size and shape matched the specs of the digital file. Silicone was infused into the porous scaffold, resulting in a soft, flexible, anatomically accurate model **(Figure 1A)**.

### Creation of the pericardial “sock”

A balloon-in-a-balloon design was used to create a latex pericardial sac model **(Figure 1B)**. First, a 45-cm latex balloon was cut transversely in half across its long axis. This was stretched tightly around the heart model from the cardiac apex to the root to mimic the visceral pericardium. Then, another 45-cm latex balloon was cut similarly and stretched over the simulated visceral pericardium to create the parietal pericardium. The free edge of the parietal pericardium was fixed using a strong glue to the visceral pericardium, creating a pericardial “sock” that is to be pulled over the ventricular chambers of the 3D heart model during assembly. A Luer-lock attachment port was incorporated into the balloon assembly to facilitate filling of the simulated pericardial space.

### Simulating the thorax using a gelatin block

A gelatin block to mimic the thoracic wall was cast as follows: 4 L of water was mixed with 600 g of plain gelatin powder. The solution was incubated at room temperature for two hours, then heated on a stovetop to 50°C while stirring until it turned clear. The gelatin solution was then poured into a rectangular container approximately the size of a human thorax, through a strainer to remove bubbles and any remaining gelatin clumps. The gelatin was cooled in a refrigerator at 20°C for six hours to yield a block mimicking the thoracic wall; the upper right portion of the block was subsequently removed and filled with uncolored tap water to create a space for the 3D-printed heart model. A notch was carved transversely across the gelatin to indicate the anatomic landmark of the subxiphoid region **(Figure 1C)**.

### Simulator assembly

The latex pericardial “sock” was pulled over the apex of the 3D-printed heart model with the upper margin of the “sock” overlying the atrioventricular groove on the heart model. Once the simulated pericardial “sock” was in place, 300 mL of water with 0.5 mL of red food coloring was injected between the two latex pericardial layers by attaching a 50-mL syringe to the Luer-lock port. After filling the pericardial “sock,” the 3D-printed cardiac model was placed into the right upper corner of the simulated thorax, ensuring that the model was positioned in the proper anatomic orientation. The construct was then placed under surgical drapes to show only the subxiphoid region of the simulated thorax.

### Study design

**Figure 2:**
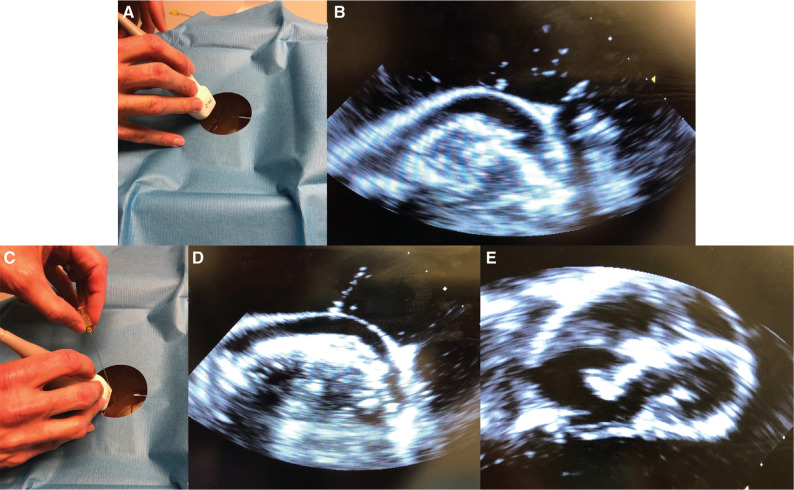
Performing mock pericardiocentesis. **A:** The model was draped to reveal only the perixiphoid area of the model. Ultrasound was used directly on the gelatin block surface with ultrasound gel. **B:** The ultrasound image visualized two cardiac ventricles. The parietal latex pericardium and the effusion between the pericardium and cardiac ventricles are visible. **C:** Under ultrasound guidance, a needle was used to puncture the gelatin block surface. The subject manipulated the ultrasound probe and needle to find the parietal pericardium. **D:** The needle can be visualized on ultrasound. Here, the needle is puncturing the latex parietal pericardium to access the pericardial effusion. The cardiac apex and ventricular septum can also be visualized. Following needle entry into the pericardial space, the effusion was drained with pericardiocentesis. **E:** Following pericardiocentesis, ultrasound confirmed resolution of the effusion. With the effusion drained, the potential pericardial space and latex parietal pericardium can no longer be seen.

Study participants consisted of pediatric interventional cardiology faculty and categorical pediatric cardiology fellows at the University of Washington. The simulator was tested over a period of two weeks at Seattle Children’s Hospital in Seattle, WA. Each subject was provided with a pericardiocentesis set and tray (Cook Medical, Bloomington, IL, USA) and instructed to perform ultrasound-guided pericardiocentesis **(Figures 2A–2E)** using a model P500 portable ultrasound machine with a P4-2 probe and the simulator (Siemens, Munich, Germany). After each procedure, participants provided verbal feedback and completed an online questionnaire. Participants rated their response to each question on a Likert scale of one (strongly disagree) to five (strongly agree) points.

### Statistical analysis

An arithmetic mean Likert score for each question was calculated from 10 responses.

## Results

**Figure 3:**
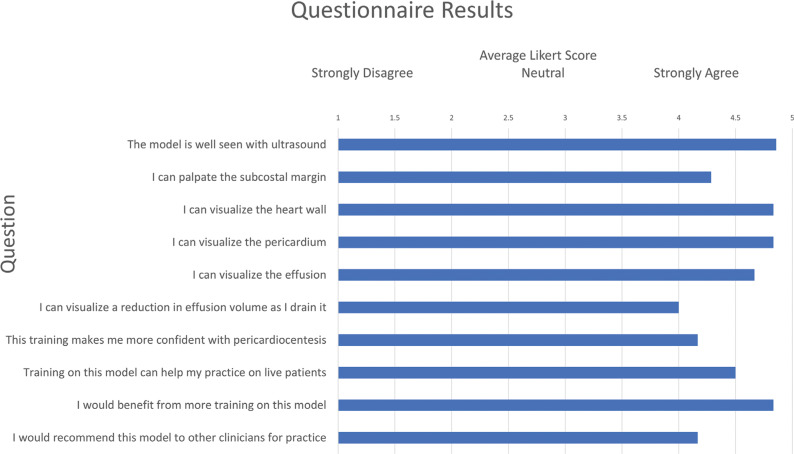
Questionnaire results from the 10 study participants. The mean Likert scores ranged from one (strongly disagree) to five (strongly agree) points for each question.

A total of 10 participants (four interventional cardiology or electrophysiology faculty and six categorical pediatric cardiology fellows) performed mock pericardiocentesis using the prototype simulator. All participants successfully inserted the needle into the pericardial space under ultrasound guidance. Following this, all but one participant successfully placed a pericardial drain using the Seldinger technique^9^ and evacuated the pericardial fluid. In all cases, the red-colored pericardial fluid was drained successfully. The resolution of the simulated pericardial effusion could be confirmed by ultrasound as the fluid was drained. All participants also completed the questionnaire, which contained questions that assessed each respondent’s impression of the model’s ultrasound compatibility, anatomical accuracy, and educational value. The mean Likert score for each question was calculated and plotted **(Figure 3)**.

## Discussion

The main contribution of this study is as a proof-of-concept investigation for a simple, inexpensive pericardiocentesis simulator that incorporates an anatomically accurate 3D-printed cardiac model and allows for mock ultrasound-guided pericardiocentesis. The model was of a relatively low cost to produce yet exhibited accurate anatomical details. It was also perceived by interventional cardiologist attendings and fellows to be a valuable educational tool. Not including the cost of the 3D-printed heart, the total cost of materials was under US$100 and both the 3D heart model and pericardiocentesis kit are reusable. Though we created our own soft 3D-printed heart model, similar products can now be purchased from several commercial vendors. The gelatin block was simple to mold and withstood multiple needle punctures and pressure from the ultrasound probe applied by up to three participants per day. After this, the gelatin block can be melted and cast again. Meanwhile, the latex pericardial sacs were switched out after each procedure. This design allows for a high-fidelity user experience while concomitantly helping academic hospitals to initiate low-cost training programs.

The principal advantage of this construct, besides its low cost and simple setup, was the incorporation of an anatomically accurate 3D heart model. By 3D printing the heart using a soft, tissue-like, ultrasound-compatible material, participants were able to visualize the cardiac walls and chambers as well as the pericardial effusion. The latex pericardial sac and effusion were also visualized on ultrasound. The red-colored effusion provided visual confirmation that the fluid was indeed being drained from the pericardial sac and not the space around the heart or from within the heart chambers. The elastic nature of the latex pericardium allowed for the visual confirmation of volume reduction as the mock effusion was drained. According to the completed questionnaires, participants generally had a positive response to the model. Most participants agreed that the model was educationally helpful. Furthermore, most believed that they could benefit from additional training using the model; notably, this trend was seen both among attendings who were experienced in pericardiocentesis and fellows who were still learning.

Baribeau et al.^8^ also recognized the need for a low-cost, high-fidelity pericardiocentesis simulator and recently reported a construct that includes a 3D-printed heart model. Their 3D heart model and method to construct the pericardium are qualitatively similar to what we have reported here. However, a fundamental difference lies in the construction of the simulator surrounding the operative field. Their model used a life-sized mannequin that was modified to accommodate the 3D-printed heart, which adds some complexity to the building process. In contrast, we formed the chest and subxiphoid regions using ultrasound-compatible gelatin. In clinical pericardiocentesis procedures, the patient is typically covered in blue drapes except for a small window located in the subxiphoid region where the ultrasound probe is placed and the needle is inserted. While palpation of the landmarks is important, this tactile feedback can be carved or molded into the gelatin. It is therefore not clear that the addition of the mannequin substantially adds to the fidelity of the simulation.

### Limitations

The goal of the present study was to conduct proof-of-concept testing for a simple and inexpensive but potentially high-fidelity pericardiocentesis simulator. As such, we did not include a direct comparison to previously reported simulators in the testing protocol. In addition, while our study addresses our simulator’s face and content validity, construct and predictive validity were not assessed in this study.

The building of our simulator with the reusable 3D-printed model was, for the most part, a simple and inexpensive process. Creating the pericardium was the most challenging aspect of the setup as this component had no corresponding 3D file or mold. Consequently, each pericardium was handmade by cutting and gluing latex balloons around the cardiac model, requiring approximately 30 minutes to construct each pericardial “sock.” A more efficient process to produce the latex pericardial sacs and effusion would be useful. We are presently working on developing a reusable mold for creating the pericardial “sock.” This could allow multiple latex pericardium models to be more quickly produced.

## Conclusions

Here, we demonstrate the feasibility and preliminary face/content validity of a simple and inexpensive pericardiocentesis simulator. Our construct combines a high-fidelity simulation using a 3D-printed heart model with a comparatively straightforward build. Looking ahead, we intend to improve the process of pericardial “sock” creation. With such modifications, we intend to further validate this construct and ultimately establish a curriculum in pericardiocentesis simulation in academic training hospitals.
